# Proliferative glomerulonephritis with monoclonal IgG deposits in an adolescent successfully treated with daratumumab

**DOI:** 10.1007/s00467-024-06425-2

**Published:** 2024-06-11

**Authors:** Eva Svabova, Jakub Zieg, Martina Sukova, Eva Flachsova, Martin Kment, Vladimir Tesar

**Affiliations:** 1 Department of Pediatrics, Second Faculty of Medicine, Charles University and Motol University Hospital, Prague, Czech Republic; 2 Department of Pediatric Hematology and Oncology, Second Faculty of Medicine, Charles University and Motol University Hospital, Prague, Czech Republic; 3 Department of Clinical and Transplant Pathology, Institute of Clinical and Experimental Medicine (IKEM), Prague, Czech Republic; 4 Department of Nephrology, First Faculty of Medicine, General University Hospital, Prague, Czech Republic

**Keywords:** Adolescent, Daratumumab, Proliferative glomerulonephritis with monoclonal IgG deposits, Proteinuria

## Abstract

There is no specific treatment for proliferative glomerulonephritis with monoclonal IgG deposits (PGNMID), a disease that is very rare in the pediatric population. We report the case of a 15-year-old boy who presented with mildly reduced kidney function and nephrotic syndrome. Kidney biopsy revealed PGNMID with monoclonal deposits of IgG3 with kappa light chain restriction. Flow cytometry showed a significant CD38 plasma cell population in the peripheral blood in the absence of other signs of hematological malignancy. The patient was treated with a 6-month course of daratumumab, a monoclonal antibody targeting CD38. There was a significant reduction in proteinuria and normalization of kidney function. Based on positive experience with adults, daratumumab should also be studied in children with PGNMID.

## Introduction

Proliferative glomerulonephritis with monoclonal IgG deposits (PGNMID) is a rare condition that mostly affects adults. Patients may present with a variable degree of proteinuria, hematuria, and reduced kidney function [1]. PGNMID may be secondary to some hematological malignancies associated with clonal proliferation of B or plasma cells, for example, multiple myeloma, but circulating monoclonal protein is found in only 30% of cases. In recent years, there has been a growing recognition that many kidney lesions are also associated with low-grade plasma cell dyscrasias or lymphoproliferative disorders. The main goal of PGNMID therapy is long-term kidney function preservation. To date, no specific treatment of PGNMID is available. Targeted therapy is used in patients with detectable B or plasma cell clones. The remaining cases are managed by various immunosuppressive agents with variable success rates. The kidney outcome is poor, with 25% of affected individuals progressing to kidney failure within 30 months [2]. There is a need for effective drugs that could significantly affect the course of the disease. Here, we present a case of an adolescent with PGNMID successfully treated with daratumumab, an anti-CD38 monoclonal antibody.

## Case presentation

**Fig. 1:**
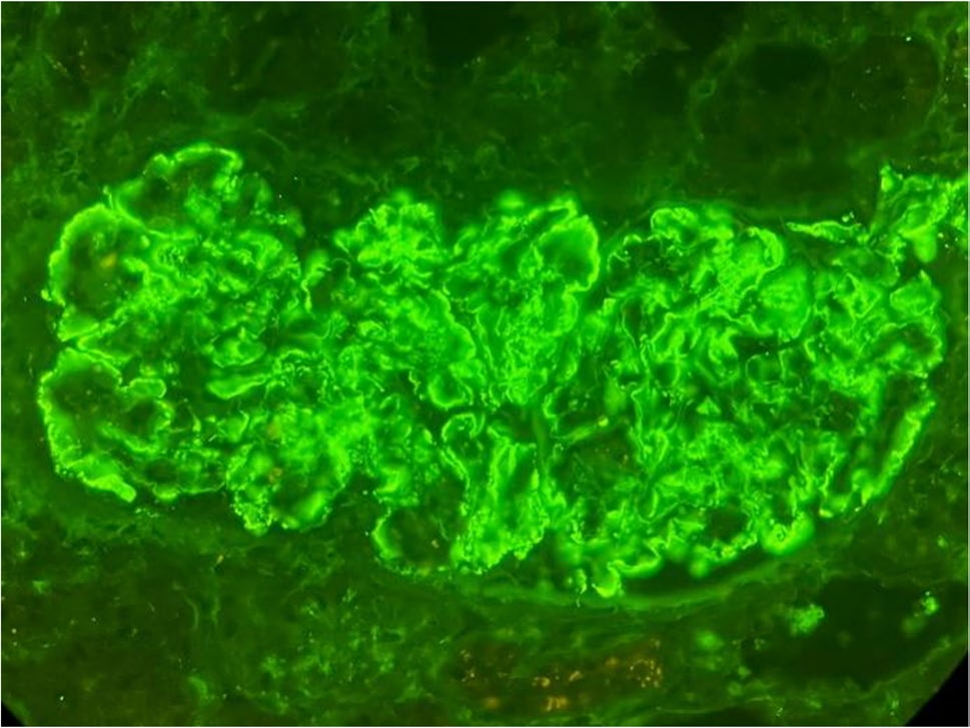
Immunofluorescence staining for IgG3. Glomerular depositions of IgG3 are evident in the peripheral capillary walls and in the mesangial area

A 15-year-old boy presented with lower limb edema and hypertension. His past medical history was unremarkable apart from atopic dermatitis. On admission, his blood pressure was 137/92 mmHg, laboratory results revealed hypoalbuminemia (serum albumin 22 g/l), creatinine 82 μmol/l corresponding to a mildly decreased glomerular filtration rate of 85 ml/min/1.73 m^2^, 3 + protein in the urine, and microscopic hematuria (86 red blood cells/ul). His urine protein to creatinine ratio (PCR) was 816 mg/mmol (upper normal limit 20 mg/mmol). His immunology results were remarkable only for a mildly lower complement C3 level of 0.74 g/l. A kidney biopsy showed membranoproliferative pattern glomerulonephritis with diffuse global mesangial and focal segmental endocapillary hypercellularity and duplication of the glomerular basement membrane. No necrosis or crescents were present. Immunofluorescence showed strong staining for IgG3 with kappa light chain restriction (Fig. 1). Electron microscopy revealed mainly mesangial deposits. Protein electrophoresis and immunofixation of serum and urine did not detect monoclonal component protein. Flow cytometry of peripheral blood showed a significant plasma cell population (30% of the B cell compartment), with high expression of CD38, but no clonal characteristics (kappa 41%, lambda 42%). There were elevated levels of free kappa and lambda chains in the urine and serum. Based on the flow cytometry results indicative of “low-plasma-cell-dyscrasia” and encouraging data from adult studies, we decided to treat our patient with daratumumab. We also added ramipril and amlodipine for hypertension and proteinuria. Initially, daratumumab infusion was administered once a week at the dose of 16 mg/kg. Then, every other week for another 8 weeks, the patient received subcutaneous injections, 1800 mg each, due to inaccessibility of intravenous daratumumab in our country. The treatment was generally well tolerated and there were no severe side effects such as anaphylaxis, hypertension, or infections. Both intravenous and subcutaneous administration of daratumumab were preceded by appropriate premedication. The patient complained of transient fatigue only on the day of drug administration, which is in agreement with the existing literature that therapy with daratumumab is associated with a manageable safety profile. This therapy led to a rapid and sustained reduction in proteinuria, which peaked only during two episodes of upper respiratory infections. Administration of daratumumab was associated with a gradual decrease of proteinuria in time; PCR 3 months after therapy initialization was 285 mg/mmol. At the end of the treatment, only mild proteinuria was present (PCR 49.5 mg/mmol) and kidney function was normal (eGFR 110 ml/min/1.73 m^2^). Control flow cytometry of peripheral blood showed 15% plasma cells, and the CD38 molecule was undetectable due to the binding of daratumumab. The concentration of kappa and lambda light chains both in serum and urine was within the normal range. Our patient also had mild normocytic normochromic anemia (hemoglobin 119 g/l) as a possible side effect of daratumumab therapy. Blood pressure was well controlled with antihypertensive therapy. During daratumumab treatment, the patient was administered anti-infectious prophylaxis with trimethoprim/sulfamethoxazole and aciclovir. The prophylaxis continued for 4 months after completion of daratumumab therapy.

## Discussion

As far as we know, this is the first pediatric case of PGNMID successfully treated with daratumumab. PGNMID occurs most often in the elderly, and the pediatric population is rarely affected [3]. Generally, there are no clear recommendations for the management of PGNMID. Therapy should be directed to the pathological clone, which is identified in only a minority of cases. Conventional immunosuppressive agents (corticosteroids and mycophenolate mofetil), the proteasome inhibitor bortezomib, and the monoclonal antibody rituximab are the commonly used drugs. However, these therapies are often associated with significant side effects and uncertain effectiveness. Daratumumab, a human IgGκ monoclonal antibody targeting CD38, is mainly used in patients with multiple myeloma. In 2021, Zand et al. [4] reported a significant sustained reduction of proteinuria and stabilization of kidney function in 10 adult patients with PGNMID. The drug was administered for 6 months and was well tolerated. Subsequently, Almaani et al. [5] analyzed five adults with bortezomib-resistant PGNMID treated with daratumumab. Four patients demonstrated some improvement—three of them achieved a kidney response, and one showed histological improvement. The good outcomes and tolerance of adult patients with PGNMID treated with daratumumab inspired us to administer this drug to our patient. Our pediatric case contributes to the existing experience with daratumumab in adults. Previous studies brought to attention that the length of therapy and frequency of daratumumab infusions are not yet clear. Additional research is needed, also in children, to assess the efficacy of daratumumab in patients with PGNMID and to identify the optimal regimen of administration.

## Summary

### What is new?

• We report a pediatric case with PGNMID successfully treated with daratumumab. Based on our experience, anti-CD38 antibody therapy should be tested in larger cohorts of children with PGNMID.

## Data Availability

The data that support the findings of this study are available on request from the corresponding author.

## References

[1] Lusco MA, Fogo AB, Najafian B, Alpers CE (2016) AJKD Atlas of Renal Pathology: proliferative glomerulonephritis with monoclonal immunoglobulin deposits. Am J Kidney Dis 67:e13–e15 26916379 10.1053/j.ajkd.2016.01.003

[2] Bridoux F, Javaugue V, Nasr SH, Leung N (2021) Proliferative glomerulonephritis with monoclonal immunoglobulin deposits: a nephrologist perspective. Nephrol Dial Transplant 36:208–215 33494099 10.1093/ndt/gfz176

[3] Xing G, Gillespie R, Bedri B, Quan A, Zhang P, Zhou XJ (2018) Proliferative glomerulonephritis with monoclonal IgG deposits in children and young adults. Pediatr Nephrol 33:1531–1538 29616329 10.1007/s00467-018-3949-8

[4] Zand L, Rajkumar SV, Leung N, Sethi S, El Ters M, Fervenza FC (2021) Safety and efficacy of daratumumab in patients with proliferative GN with monoclonal immunoglobulin deposits. J Am Soc Nephrol 32:1163–1173 33685975 10.1681/ASN.2020101541 PMC8259683

[5] Almaani S, Parikh SV, Satoskar AA, Bumma N, Rovin BH, Sharma N, Efebera Y, Ayoub I (2021) Daratumumab in patients with bortezomib-refractory proliferative glomerulonephritis with monoclonal immunoglobulin deposits. Kidney Int Rep 6:2203–2206 34386669 10.1016/j.ekir.2021.05.008 PMC8343787

